# Genome-wide methylation profiling of the bronchial mucosa of asthmatics: relationship to atopy

**DOI:** 10.1186/1471-2350-14-39

**Published:** 2013-03-24

**Authors:** Yoon-Jeong Kim, Sung-Woo Park, Tae-Hoon Kim, Jong-Sook Park, Hyun Sub Cheong, Hyoung Doo Shin, Choon-Sik Park

**Affiliations:** 1 Division of Allergy and Respiratory Medicine, Department of Internal Medicine, Soonchunhyang University Bucheon Hospital, 1174, Jung Dong, Wonmi-Gu, Bucheon, Gyeonggi Do 420-021, Korea; 2 Department of Genetic Epidemiology, SNP Genetics, Inc., Room 1407, 14th floor, B-dong, WooLim Lion’s Valley, 371-28, Gasan-dong, Geumcheon-Gu, Seoul 153-803, Korea; 3 Department of Life Science, Sogang University, 1 Shinsu-dong, Mapo-gu, Seoul 121-742, Korea

**Keywords:** Asthma, Bronchial mucosa, Atopy, DNA methylation, Epigenetics, Environmental factor

## Abstract

**Background:**

Asthma is a common respiratory disease that is characterized by bronchial hyperresponsiveness and airway obstruction due to chronic airway inflammation. Atopic asthma is a typical IgE-mediated disease in which the enhanced production of IgE is driven by the activation of Th2 cells, which release a distinct pattern of cytokines, including interleukin 4 (IL4) and IL3, in response to specific antigen presentation. To evaluate the methylation status of the whole genomes of bronchial mucosa tissues from subjects who lacked or had sensitization to *Dermatophagoides farina* (*Df*) and *Dermatophagoides pteronyssinus* (*Dp*).

**Methods:**

The genome-wide DNA methylation levels in the bronchial mucosa tissues of atopic asthmatics (N = 10), non-atopic asthmatics (N = 7), and normal controls (N = 7) were examined using microarrays.

**Results:**

In the bronchial mucosa of atopic asthmatics, hypermethylation was detected at 6 loci in 6 genes, while hypomethylation was detected at 49 loci in 48 genes compared to those of non-atopic asthmatics. Genes that were assigned the ontologies of multicellular organismal process, response to organic substance, hormone metabolic process, and growth factor receptor binding were hypomethylated. The methylation levels in the mucosa of asthmatics and normal controls were similar.

**Conclusions:**

The bronchial mucosa of asthmatics who are atopic to *Df* or *Dp* have characteristic methylation patterns for 52 genes. The genes and pathways identified in the present study may be associated with the presence of atopy in asthmatics and therefore represent attractive targets for future research.

## Background

Asthma is a common respiratory disease that is characterized by bronchial hyperresponsiveness and airway obstruction due to chronic airway inflammation [1]. These processes are attributed to the increased expression of genes for several inflammatory and immune mediators, including cytokines, chemokines, adhesion molecules, enzymes, and chemical mediators [2]. The complex traits that result from interactions between multiple disease susceptibility factors and the surrounding environment have major influences on the onset and severity of asthma [3].

Atopic asthma is a typical IgE-mediated disease in which the enhanced production of IgE is driven by the activation of Th2 cells, which release a distinct pattern of cytokines, including interleukin 4 (IL4) and IL3, in response to specific antigen presentation [4,5]. Atopy, which is characterized by the presence of IgE specific for inhalant allergens or by high-level production of IgE, is closely associated with asthma development [6]. The prevalence of asthma has increased throughout the world over the past three decades [7]. However, wide variations have been noted among populations of the same races, indicating different exposures to environmental factors [8]. Environmental factors during the pre- and post-natal periods, including the extent of allergens such as obesity, and exposure to smoking and air pollutants are risk factors for asthma. Furthermore, asthma is triggered and exacerbated by outdoor allergens and air pollutants, including pollens, and microbial and viral pathogens [9-11]. It is noteworthy that many of these indoor and outdoor inducers/triggers of asthma program the immature airway during early life, without any effect on the host DNA, leading to altered asthma risk in later life [12]. Epigenetics consists of changes in gene expression due to mechanisms other than changes to the underlying DNA sequence [13,14]. These changes may induce phenotypic change and persist through cell divisions for the remainder of the cell's life, possibly lasting for multiple generations without any change in the intrinsic DNA sequence of the organism. Epigenetic changes include histone deacetylation, DNA methylation and non-coding RNAs. The DNA methylation is the covalent addition of a methyl group to a cytosine residue in a CpG dinucleotide [15]. Regions of methylated DNA have been correlated with the tissue-specific expression of several genes and with active coding regions across the genome [16]. Recently, transitory methylation has been demonstrated for specific gene regulatory regions [17]. DNA methylation analysis can be grouped into the mutually exclusive categories of annotated promoters (15%), untranslated regions (UTRs; 7%), exons (13%), introns (31%), and intergenic segments (34%) according to each regions [18]. However, the global distribution of DNA methylation in human bronchial mucosa tissue is unknown, and its role in the development of asthma has not been reported to date.

The airway inflammation and remodeling that occur in asthma are characterized by the infiltration of lymphocytes and eosinophils into the bronchial mucosa, accompanied by structural changes, which include epithelial desquamation, subepithelial fibrosis, mucus gland hyperplasia, modification of the extracellular matrix, and hypertrophy/hyperplasia of smooth muscle cells [19,20]. Thus, bronchial mucosa tissues are suitable for studying the role of epigenetics in airway responses to environmental factors, such as indoor allergens. In the present study, using microarray analyses, we analyzed the genome-wide DNA methylation profiles of bronchial mucosa tissues from two groups of asthmatics who lacked or carried specific IgE to the dust mites *Dermatophagoides farina* (*Df*) and *Dermatophagoides pteronyssinus* (*Dp*), and from normal controls, to reveal the epigenetic differences between house dust mite-sensitized subjects and naïve subjects.

## Methods

### Subjects

All samples were obtained after an informed, written consent was obtained from each patient and the protocol was approved by the local ethic committee (The ethics committee's approval number: SCHBC-IRB-2005-02). The bronchial mucosa tissues from atopic asthmatics for *Df* and *Dp* (N = 10), non-atopic asthmatics (N = 7), and normal controls (N = 7) were obtained from the Biobank after the experimental protocol was approved by the Ethics Committee in the biobank of Soonchunhyang University Hospital (Schbc-biobank-2011-004). All patients were diagnosed by physicians and met the definition of asthma provided in the Global Initiative for Asthma (GINA) guidelines [1]. All patients had a history of dyspnea and wheezing during the previous 12 months, plus one of the following: 1) >15% increase in forced expiratory volume in 1 second (FEV_1_) or >12% increase plus 200 mL following inhalation of a short-acting bronchodilator; 2) <10 mg/mL PC20 methacholine; or 3) >20% increase in FEV_1_ following 2 weeks of treatment with inhaled or systemic corticosteroids. The normal subjects, who were recruited from the spouses of the patients and from the general population, answered negatively to a screening questionnaire for respiratory symptoms [21] and had an FEV1 *>*75% of the predicted value, PC20 methacholine *>*10 mg/ml, and normal findings on a plain chest X-ray.

### Allergy skin prick tests and specific IgE measurements

Allergy skin prick tests were performed using 24 commercial inhalant allergens, which included dust mites (*Df* and *Dp*), fungi, cat fur, dog fur, cockroaches, and grass, tree, and weed pollens (Bencard Co. Ltd., Brentford, UK). All of the tests included positive (1 mg/mL histamine) and negative (diluent) controls. Atopy was defined as having a wheal reaction equal to or greater than 3 mm in diameter or equal to or greater than that induced by histamine. IgE specific for *Df* and *Dp* was measured using the CAP system (Pharmacia Diagnostics, Uppsala, Sweden). The concentrations were measured in kU/l and expressed semi-quantitatively as classes 0–6. Sensitization to house dust mites was confirmed by a positive skin prick test to house dust mites and elevated allergen-specific IgE level (greater than score 2). Non-atopy was defined as a negative skin prick test to all allergens tested and a negative result for specific IgE (score 0) raised in response to *Df* and *Dp*.

### Genomic DNA isolation

All the mucosal biopsies had been taken from the segmental carinae of the right lower lobe, fixed in 4% buffered formaldehyde solution, and embedded in paraffin blocks. DNA was purified from the tissues using the QIAamp DNA Blood Mini Kit (Qiagen, Crawley, UK).

### Bisulfite conversion

Bisulphite conversion of genomic DNA was done with the EZ DNA methylation Kit (Zymo Research, Irvine, CA, USA) by following the manufacturer's protocol with modifications for the Illumina Infinium Methylation Assay. Briefly, one microgram of genomic DNA was first mixed with 5 μl of M-Dilution Buffer and incubated at 37°C for 15 minutes and then mixed with 100 μl of CT Conversion Reagent prepared as instructed in the protocol. Mixtures were incubated in a thermocycler for 16 cycles at 95°C for 30 seconds and 50°C for 60 minutes. Bisulphite-converted DNA samples were loaded onto 96-column plates provided in the kit for desulphonation and purification as instructed in the protocol.

### DNA methylation assay and differential DNA methylation analysis

Bisulphite-converted genomic DNA was analyzed using Human Methylation27 Beadchip (Illumina, San Diego, CA, USA). The beadchip contains 27,578 CpG loci covering more than 14,000 human RefSeq genes at single-nucleotide resolution. Chip process and data analysis were performed by using reagents provided in the kit and by following the manufacturer's manual. Briefly, 4 μl of bisulphite-converted genomic DNA was denatured in 0.014 N sodium hydroxide, neutralized and amplified with kit-provided reagents and buffer for 20–24 hours at 37°C. Samples were fragmented. 12 μl of each sample was loaded onto a 12-sample chip and the chips were assembled into a hybridization chamber as instructed in the manual. After incubation at 48°C for 16–20 hours, chips were washed with wash buffers provided in the kit and assembled and placed into a fluid flow-through station for primer-extension reaction and staining with reagents and buffers provided in the kit. Polymer-coated chips were image-processed in Illumina's iScan scanner. Data were extracted using GenomeStudio software. Methylation values for each CpG locus are expressed as a beta (β)-value, representing a continuous measurement from 0 (completely unmethylated) to 1 (completely methylated). This value is based on following definition and calculation: β-value = (signal intensity of methylation-detection probe)/(signal intensity of methylation- detection probe + signal intensity of non-methylation-detection probe). The intensity data were normalized using the background function.

### Statistics analysis

To identify differentially methylated CpG sites between the groups, the difference in mean methylation levels between the two groups was analyzed using a two-sided *t*-test. Assuming a normal distribution of β-value, DeltaBeta (|Δβ|) was calculated as β for the bronchial asthma (BA) group minus that for the normal control (NC) group or atopic asthmatics minus that for the non-atopic ones. Selection of the differentially methylated CpG loci between groups was based on (1) a β-value difference of >0.10 and (2) a P-value of <0.01. Heatmap of the differentially methylated CpG sites was done and viewed by GenomeStudio software. Gene ontology enrichment of DNA methylation profile was performed using GOTM (http://bioinfo.vanderbilt.edu/gotm/), which is based on hypergeometric test to show the overrepresented gene ontology categories (p-value < 0.01) [22]. The p-value was also calculated according to BINOMDIST function on the basis of the overrepresentation of gene ontology categories in hypermethylated and hypomethylated genes when comparing to all genes on the chip, as a confirmation of the significance of results.

Statistical analyses were performed with SPSS 10.0. The Mann–Whitney test (two-sample rank-sum test) was used to analyze differences between two groups. All data are expressed as median values and interquartile ranges; significance was defined as *P* <0.05.

## Results

### Clinical characteristics of the study subjects

The 17 patients with asthma were classified into two groups (atopic and non-atopic to *Df* or *Dp*) based on the results of the skin tests and assays for specific IgE to *Df* and *Dp*. The characteristics of the asthmatics and normal controls are summarized in Table 1. There were no significant differences in terms of age, sex, smoking ratio, and FEV1 between the atopic and non-atopic patients. The total IgE concentration and the level of specific IgE to *Df* and *Dp* were significantly higher in the atopic group than in the non-atopic group (*P* < 0.03, and *P* < 0.001, respectively).

**Table 1:** Clinical characteristics of patients with bronchial mucosa tissues for DNA Methylation chip

**Clinical profile**	**NC**	**Atopic asthmatics**	**Non atopic asthmatics**	**P-value**	
				**NC vs BA**	**in BA**
Number	7	10	7		
Age, years (range)	50 (22–71)	35 (21–60)	47 (28–78)	0.288	0.133
Sex, male, n(%)	1 (14.3)	4 (40)	1 (14.3)	0.629	0.338
exsmoker/nonsmoker (%)	0 / 7	2 / 8	1 / 6	0.299	0.677
Positive skin test rate (%)*	ND	100*	0	**-**	**0.00005**
FVC (% predicted)	89.20 ± 10.18	79.50 ± 16.89	90.80 ± 22.26	0.349	0.371
FEV_1_ (% predicted)	102.00 ± 16.84	78.50 ± 22.54	90.60 ± 20.77	0.119	0.44
FEV_1_/FVC (% predicted)	85.80 ± 6.18	77.00 ± 15.12	76.20 ± 6.10	0.168	0.513
Body mass index (kg/m2)	23.37 ± 4.08	25.31 ± 1.66	25.92 ± 3.20	0.497	0.37
Total IgE (IU/M)	37.2	663.95 ± 784.99	111.28 ± 99.90	0.267	**0.036**
*Dp.* specific IgE (score)	ND	4 (2–6)	0	-	**0.0005**
*Df.* specific IgE (score)	ND	4 (2–6)	0	-	**0.0005**

Data are presented as mean ± standard deviation or as median (range).

*Positive skin prick test to house dust mites(*D. pteronyssinus, or D. farina*)*.*

BA:Bronchial asthma; NC:Normal control.

### Distribution of DNA methylation levels among the three groups

**Figure 1:**
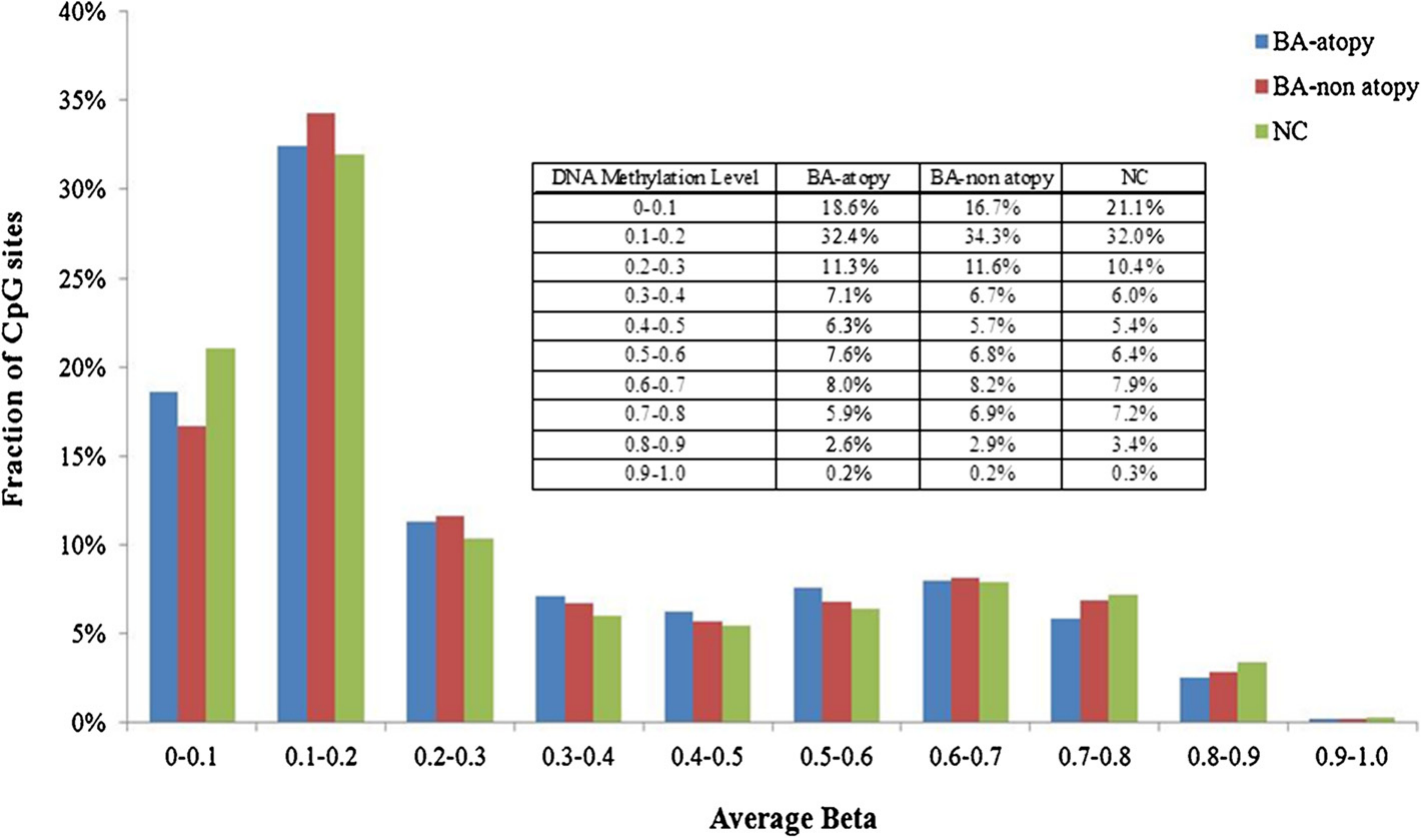
**Distribution of the DNA methylation levels of BA-atopy, non atopy and NC in bronchial mucosa.** Average beta: DNA methylation level (0–1).

DNA methylation levels were measured with Illumina’s genome-wide methylation assay chip of 27 K CpG sites. Methylation of the X-chromosome was not considered in this analysis. Of the 27578 CpG sites, 14363 were located within promoter regions, while 13215 loci were in the gene body. As shown in Figure 1, the methylation levels in the three groups showed a similar unimodel distribution, with the highest peak on the *x*-axis (average β, 0.1–0.2) and the lowest peak at the right end (average β, 0.9–1.0). Approximately 50% of the loci had an average β value <0.2, whereas 0.3% of the loci had an average β value of 0.9–1.0.

### Comparison of DNA methylation patterns of the BA and NC groups

**Figure 2:**
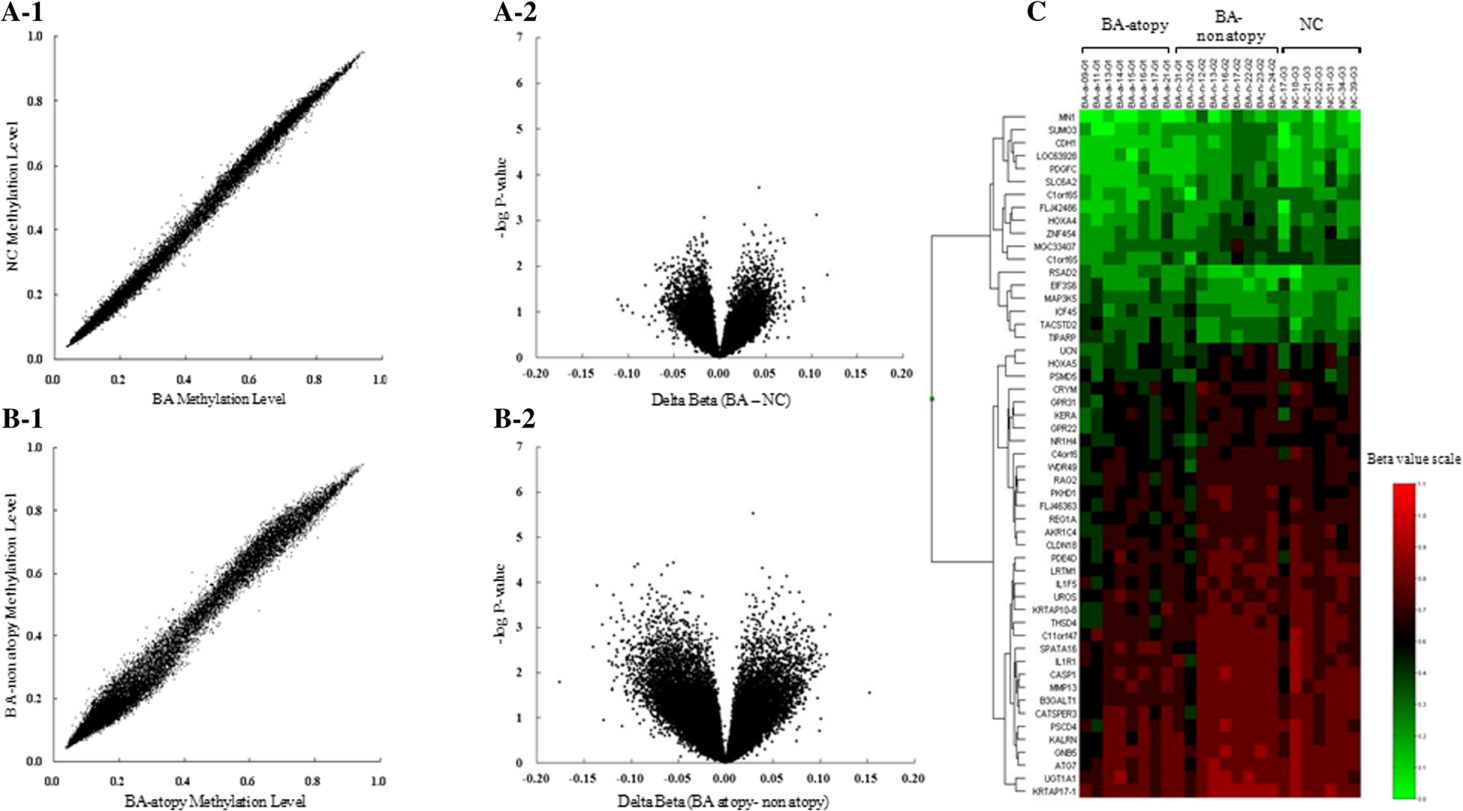
**Summary of DNA methylation data.** (**A-1**) Scatter plot of similar methylation levels between BA and NC. (**A-2**) Volcano plot of the |Db| values between BA and NC. (**B-1**) Scatter plot of similar methylation levels between BA-atopy and non atopy. DeltaBeta: difference of DNA methylation levels. (**B-2**) Volcano plot of the |Db| values between BA-atopy and non atopy. log(P): log-transformed t-test P-values. (**C**) Heat map of 53 methylated CpGs on whole chromosome between the three groups (BA-atopy, BA-non atopy, NC). The DNA methylation profiles were similar between the three groups.

The methylation levels were similar between the mucosa of the asthmatics and the normal controls (Figure 2A). The |Δβ| values for the methylation levels were calculated as the average β value for the NC group minus that for the BA group. A volcano plot of the |Δβ| values for each locus in the bronchial mucosa tissues is shown in Figure 2A-2. The values ranged from −0.2 to 0.2. Although the DNA methylation levels were not remarkably different between the two groups (Figure 2A-1), one methylation site in a CpG motif in the promoter of *LCN6* showed a significant difference (*P* < 0.01) in |Δβ| value (0.11) between the two groups (Table 2).

**Table 2:** DNA methylation level of CpGs in genes between BA and NC

							**BA vs NC**				
N	**TargetID**	**Gene name**	**Chr**	**CpG**	**Position**	**CpG**	**Beta value of**	**Beta value of**	**DeltaBeta**	**P-value**	**FDR q-value**
				**Coordinate**		**Island**	**BA (M ± SD)**	**NC (M ± SD)**	**(NC-BA)**		
1	cg14611112	LCN6	9	138762801	Promoter	FALSE	0.46 ± 0.07	0.56 ± 0.04	0.11	0.0008	0.005

### Comparison of DNA methylation patterns between asthmatics atopic or non-atopic for *Df* and *Dp*

The DNA methylation profiles were similar for the two groups, with a narrow range of |Δβ| values from −0.2 to 0.2. A volcano plot of the |Δβ| values for each locus in the bronchial mucosa tissues is shown in Figure 2B-2. To identify the loci that showed atopy-specific changes in methylation, we applied the following criteria: *P* ≤ 0.01 and |Δβ| ≥ 0.10. Six loci in six genes in the bronchial mucosa samples showed significant increases in methylation among the atopic patients, as compared to the non-atopic patients, whereas 47 loci in 46 genes showed significant decreases in methylation in these samples (Table 3). Heat mapping of the β values for the 53 loci revealed distinct DNA methylation profiles for the bronchial mucosa tissues of the atopic and non-atopic groups (Figure 2C). Among these loci (n = 53), 24 were located within CpG islands, while 29 were outside CpG islands. In all, 31 loci were identified within promoter regions (2 kb from the transcriptional start site), while 22 loci were in the gene body (Table 3).

**Table 3:** DNA methylation level of CpGs in genes between BA-atopy and non atopy

							**BA atopy vs BA non atopy**				
**N**	**TargetID**	**Gene name**	**Chr**	**CpG Coordinate**	**Position**	**CpG Island**	**Beta value of BA atopy (M ± SD)**	**Beta value of BA non atopy (M ± SD)**	**DeltaBeta (BA atopy-BA non atopy)**	**P-value**	**FDR q-value**
1	cg15804973	MAP3K5	6	137155349	Promoter	TRUE	0.37 ± 0.06	0.26 ± 0.04	0.11	0.0005	-
2	cg18201077	RSAD2	2	6935359	Promoter	FALSE	0.29 ± 0.06	0.19 ± 0.03	0.11	0.001	0.005
3	cg24262469	TIPARP	3	157875073	Promoter	TRUE	0.42 ± 0.08	0.31 ± 0.03	0.11	0.004	-
4	cg23123362	ICF45	5	157091024	Promoter	FALSE	0.39 ± 0.07	0.29 ± 0.03	0.1	0.004	0.007
5	cg06987504	EIF3S6	8	109330128	Promoter	TRUE	0.33 ± 0.08	0.22 ± 0.04	0.1	0.007	-
6	cg27398499	TACSTD2	1	58816033	Promoter	FALSE	0.41 ± 0.08	0.31 ± 0.05	0.1	0.009	0.009
7	cg23989635	CDH1	16	67328696	Gene body	TRUE	0.16 ± 0.03	0.29 ± 0.07	−0.14	0.0001	-
8	cg25368651	C11orf47	11	6475107	Promoter	TRUE	0.64 ± 0.06	0.76 ± 0.02	−0.12	0.0002	-
9	cg25427580	B3GALT1	2	168383428	Gene body	TRUE	0.63 ± 0.06	0.74 ± 0.03	−0.11	0.0003	-
10	cg18885346	PKHD1	6	52060382	Promoter	FALSE	0.57 ± 0.06	0.68 ± 0.04	−0.11	0.0004	0.013
11	cg15309006	LOC63928	16	23673449	Gene body	TRUE	0.16 ± 0.05	0.27 ± 0.05	−0.11	0.0005	-
12	cg09272256	AKR1C4	10	5228798	Gene body	FALSE	0.56 ± 0.07	0.68 ± 0.03	−0.12	0.0006	0.010
13	cg11532513	LRTM1	3	54937112	Promoter	FALSE	0.60 ± 0.05	0.71 ± 0.05	−0.11	0.0007	0.007
14	cg16242770	KRTAP17-1	17	36725473	Gene body	FALSE	0.71 ± 0.06	0.82 ± 0.02	−0.1	0.0007	0.006
15	cg00051623	CASP1	11	104411067	Promoter	FALSE	0.65 ± 0.07	0.76 ± 0.03	−0.12	0.0008	0.004
16	cg20587168	MGC33407	19	8670172	Gene body	TRUE	0.33 ± 0.06	0.46 ± 0.07	−0.13	0.0009	-
17	cg03355526	ZNF454	5	178300830	Gene body	TRUE	0.28 ± 0.06	0.39 ± 0.05	−0.11	0.001	-
18	cg24648061	GPR22	7	106901742	Gene body	FALSE	0.50 ± 0.07	0.62 ± 0.04	−0.12	0.002	0.007
19	cg11237738	C4orf6	4	5577784	Gene body	FALSE	0.54 ± 0.07	0.64 ± 0.02	−0.1	0.002	0.006
20	cg03734874	FLJ42486	14	104142142	Promoter	TRUE	0.23 ± 0.06	0.34 ± 0.05	−0.11	0.002	-
21	cg20937139	PDGFC	4	158111996	Promoter	TRUE	0.17 ± 0.06	0.28 ± 0.06	−0.11	0.002	-
22	cg26657648	IL1R1	2	102136834	Promoter	FALSE	0.64 ± 0.07	0.75 ± 0.03	−0.11	0.002	0.006
23	cg23111544	REG1A	2	79201092	Promoter	FALSE	0.57 ± 0.07	0.67 ± 0.03	−0.1	0.002	0.005
24	cg07823755	UGT1A1	2	234333658	Promoter	FALSE	0.71 ± 0.07	0.81 ± 0.03	−0.1	0.002	0.005
25	cg13112511	PDE4D	5	58918032	Promoter	FALSE	0.59 ± 0.09	0.71 ± 0.03	−0.12	0.002	0.005
26	cg21717724	PSMD5	9	122645027	Gene body	TRUE	0.47 ± 0.07	0.59 ± 0.06	−0.12	0.002	-
27	cg08509737	ATG7	3	11315160	Gene body	FALSE	0.68 ± 0.07	0.78 ± 0.02	−0.1	0.002	0.004
28	cg05333568	C1orf65	1	221633391	Gene body	TRUE	0.29 ± 0.08	0.43 ± 0.08	−0.14	0.003	-
29	cg16398289	WDR49	3	168853983	Promoter	FALSE	0.56 ± 0.08	0.66 ± 0.03	−0.11	0.003	0.006
30	cg04527918	UCN	2	27384634	Promoter	TRUE	0.42 ± 0.06	0.54 ± 0.08	−0.12	0.003	-
31	cg20088913	FLJ46363	12	9997203	Promoter	FALSE	0.57 ± 0.07	0.67 ± 0.03	−0.1	0.003	0.005
32	cg10430690	KALRN	3	125296275	Gene body	TRUE	0.65 ± 0.09	0.77 ± 0.02	−0.11	0.004	-
33	cg15381313	NR1H4	12	99391810	Promoter	FALSE	0.50 ± 0.06	0.60 ± 0.07	−0.1	0.004	0.006
34	cg17778867	KRTAP10-8	21	44856424	Gene body	FALSE	0.60 ± 0.09	0.71 ± 0.02	−0.12	0.004	0.006
35	cg01216369	SPATA16	3	174341695	Promoter	TRUE	0.64 ± 0.08	0.74 ± 0.02	−0.1	0.005	-
36	cg01507173	IL1F5	2	113532686	Gene body	FALSE	0.59 ± 0.08	0.69 ± 0.02	−0.1	0.005	0.007
37	cg22152192	CRYM	16	21221918	Promoter	FALSE	0.52 ± 0.08	0.63 ± 0.04	−0.1	0.005	0.007
38	cg21053323	SUMO3	21	45062472	Gene body	TRUE	0.20 ± 0.07	0.30 ± 0.06	−0.1	0.005	-
39	cg23324787	RAG2	11	36576362	Gene body	FALSE	0.57 ± 0.08	0.67 ± 0.03	−0.1	0.005	0.007
40	cg13041032	MMP13	11	102331672	Gene body	FALSE	0.64 ± 0.08	0.74 ± 0.03	−0.1	0.005	0.006
41	cg10784090	CLDN18	3	139200348	Promoter	TRUE	0.59 ± 0.08	0.70 ± 0.04	−0.1	0.005	-
42	cg04317399	HOXA4	7	27136924	Gene body	TRUE	0.26 ± 0.07	0.38 ± 0.09	−0.12	0.006	-
43	cg27202708	C1orf65	1	221633391	Promoter	TRUE	0.22 ± 0.07	0.34 ± 0.08	−0.12	0.006	-
44	cg20300655	CATSPER3	5	134331513	Promoter	FALSE	0.64 ± 0.08	0.74 ± 0.02	−0.1	0.006	0.007
45	cg10094277	KERA	12	89976262	Gene body	FALSE	0.51 ± 0.09	0.62 ± 0.03	−0.11	0.007	0.008
46	cg04117029	UROS	10	127501757	Promoter	FALSE	0.60 ± 0.09	0.71 ± 0.03	−0.11	0.007	0.008
47	cg12588299	GPR31	6	167491309	Promoter	FALSE	0.50 ± 0.08	0.61 ± 0.05	−0.11	0.007	0.008
48	cg27269921	MN1	22	26527486	Gene body	TRUE	0.11 ± 0.03	0.23 ± 0.12	−0.12	0.007	-
49	cg10362591	SLC6A2	16	54248057	Promoter	TRUE	0.21 ± 0.06	0.33 ± 0.09	−0.12	0.007	-
50	cg02248486	HOXA5	7	27149812	Gene body	TRUE	0.47 ± 0.07	0.57 ± 0.07	−0.1	0.008	-
51	cg04616566	THSD4	15	69807942	Promoter	FALSE	0.60 ± 0.09	0.72 ± 0.03	−0.11	0.008	0.009
52	cg19366178	GNB5	15	50270857	Promoter	FALSE	0.69 ± 0.09	0.79 ± 0.02	−0.1	0.008	0.008
53	cg05259765	PSCD4	22	36008370	Promoter	TRUE	0.66 ± 0.08	0.77 ± 0.03	−0.1	0.009	-

### Gene ontology analysis of the differentially methylated genes in asthmatics atopic or non-atopic for *Df* and *Dp*

To examine the biological functions associated with the genes that had significantly hypomethylated and hypermethylated loci, we analyzed the gene ontologic categories of 52 hypomethylated and hypermethylated genes using the GOTM program (http://bioinfo.vanderbilt.edu/gotm/) [22]. The genes present on the Illumina methylation assay chip were used as a reference list. The genes with hypomethylated loci were assigned to the distinct gene ontologic categories of multicellular process, response to organic substance, hormone metabolic process, and growth factor receptor binding (Table 4 and Additional file 1: Table S2).

**Table 4:** Gene ontology analysis of genes with different methylation states according to atopic status among BA

**Methylation states**	**Category**	**Gene ontology category**	**Genes**	**Observed gene number**	**Expected gene number**	**Ratio of enrichment**	**Significance of enrichment**
Hypomethylation	Biological process	Cellular hormone metabolic process	UGT1A1, CRYM, AKR1C4	3	0.21	14.37	0.001
		Positive regulation of DNA replication	PDGFC, UCN	2	0.08	25.06	0.003
		Response to organic substance	IL1R1, UGT1A1, UROS, CDH1, CASP1, NR1H4, UCN	7	1.97	3.55	0.003
		Porphyrin metabolic process	UGT1A1, UROS	2	0.09	23.27	0.003
		Multicellular organismal process	UGT1A1, PDGFC, KERA, C4orf6, CATSPER3, CRYM, RAG2, NR1H4,KALRN, PKHD1, CLCN5, UCN, CDH1, HOXA4, HOXA5, PDE4D, MMP13, CASP1, SLC6A2	19	10.87	1.75	0.004
		Monoamine transport	CRYM, SLC6A2	2	0.11	18.62	0.005
		Collagen metabolic process	MMP13, UCN	2	0.12	16.71	0.006
	Molecular function	Growth factor receptor binding	IL1R1, PDGFC, IL1F5	3	0.20	14.83	0.001
	Cellular component	Extrinsic to plasma membrane	GNB5, CDH1	2	0.14	14.75	0.008
Hypermethylation	Biological process	Signal transduction	MAP3K5, TACSTD2	2	0.13	15.73	0.005
		Immune response	RSAD2	1	0.05	14.96	0.001
		metabolic process	TIPARP	1	0.05	13.49	0.004
	Molecular function	Protein kinase activity	MAP3K5	1	0.04	22.48	0.002
		DNA binding	TIPARP	1	0.05	18.76	0.004
		Receptor activity	TACSTD2	1	0.03	14.64	0.009
	Cellular component	Cell surface receptor	TACSTD2	1	0.04	14.59	0.009

P-values of enrichment significance below 0.01 were shown.

## Discussion

DNA methylation is essential for cell differentiation and development. Moreover, for many years, DNA methylation has observed to play a role in regulating gene expression. Methylation of gene promoter region is correlated with low or no transcription [23]. Recent research revealed novel insights on the location of DNA hypermethylation. Hypermethylation of intra- and intergenic CpG dinucleotides might contribute to regulating gene expression by functioning as alternative promoters [24]. For example, in-depth investigation of the human SHANK3 locus (~60 kb) showed hypermethylation-regulated intragenic promoter activity, expressing alternative transcripts in a tissue- (brain) and cell-type (primary cortical astrocyte)–specific manner [24]. In addition, other gene-regulating regions such as enhancers, which are *cis*-regulatory DNA sequences that increase transcription independent of their orientation and distance relative to the TSS, can be regulated by hypermethylation [25,26]. For example, hypermethylation-dependent enhancer-like activity, located at a CpG island in EGFR2 intron 1, is suggested to regulate transcription [27]. In the present study, the methylation levels in the three sample groups had an average β value that ranged from 0 to 0.2 for 50% of the loci on the entire methylome. Continuously expressed genes, including housekeeping genes, may be included in this group (Additional file 1: Table S3). The lowest peak, representing about 0.2% of the loci, was at the right end (average β, 0.9–1.0). These genes may be densely packed in nucleosomes. The methylation levels in the three sample groups showed a similar pattern. However, recent studies also identified positive associations between expression and methylation level [28-30]. Further gene expression analysis can be integrated with this DNA methylation data, and can reach about the exact role of DNA methylation in controlling gene expression.

Comparing the average β value for each locus in the bronchial mucosa tissues of the asthmatics atopic to house dust mites with those of the non-atopic asthmatics, 47 loci in 46 genes showed significant decreases in methylation, while 6 loci in 6 genes showed relative increases in methylation. These data suggest that the phenotypic differences in atopic sensitization to house dust mites originate from differences in gene regulation in the lower airways. Notably, the hypomethylated genes were linked to multicellular process, hormone metabolic process, response to organic substance, and growth factor receptor binding. Changes in the expression levels of these genes caused by hypomethylation might lead to sensitization to inhaled allergens, such as house dust mites or pollens.

In the present study, we did not examine the levels of mRNA expression in the bronchial mucosa of the subjects, since the mucosal biopsy samples were too small to extract DNA and RNA at the same time. However, one interesting finding is decrease of the methylation for mitogen-activated protein kinase 5 (*MAP3K5*) gene in non-atopic asthmatics compared to asthmatics atopic for *Dp*. The intronic SNP rs9494554, located within MAP3K5, was associated with atopy in the pooling and replication samples, and remained significant in the combined analysis after Bonferroni correction. Other SNP in MAP3K5 (rs9402839) was nominally significant for atopy in pooling sample and border significant in the replication step. MAP3K5 has been found to be associated with atopy [31]. *MAP3K5* encodes a member of the mitogen-activated protein kinase family that regulates the activation of the transcription factor activator protein-1(AP1) in leukotriene D4-stimulated airway smooth muscle cells and in nitric oxygen-stimulated bronchial epithelial cells [32,33].

E-cadherin (CDH1) gene polymorphisms are associated with airway remodelling, inflammation and lung function decline in individuals with asthma. CDH1 SNPs are associated with epithelial E-cadherin expression and suggest that epithelial adhesion is an important contributor to airway remodeling and lung function in asthma [34].

In bronchial epithelial cells, Der p 2, which is a major non-proteolytic allergen of *Dp*, induced dose-dependent up-regulation of both mRNA expression and protein secretion of T cell-directed CC chemokine, granulocyte-macrophage colony-stimulating factor, IL6, IL8, monocyte chemotactic protein-1, and macrophage inflammatory protein-3a *via* NF-κB and MAPK activation [35,36]. However, many of the genes identified in the present study have not been linked with atopy to *Dp*. Thus, further functional characterization of these genes is needed to clarify the relationship between the methylation of these genes and the development of atopy. These mechanisms include genomic imprinting, histone modification, altered DNA methylation of regulatory sequences, which may change asthma risk after conception via environmentally mediated epigenetic disruption of gene expression [37,38]. Since the pattern of CpG island methylation is generally cell line-specific, the sampled airway mucosa should not differ in cellular composition between the groups. Previous studies comparing pathologic findings demonstrated that the histologic appearance of the bronchial mucosa from atopic asthmatics did not differ from that of non-atopic asthmatics [39]. Although we did not perform differential counts of the infiltrating cells and the cells comprising the bronchial mucosa, the extent of cellular infiltration, as assessed by hematoxylin and eosin staining, was similar in the two groups (data not shown). However, previous immunohistochemical studies of bronchial mucosa have shown different expression patterns of adhesion molecules, cytokines, chemokines, inflammatory mediators, and their receptors [40,41]. Therefore, we consider that the difference in methylation patterns is associated with different stages of cellular differentiation or activation, rather than the cellular composition of the bronchial mucosa. The present study confirms the existence of atopy-associated methylation in bronchial mucosa tissues. The bronchial mucosa samples from asthmatics with atopy show characteristic methylation patterns for 52 genes. In addition Limitations of this study are the relatively small sample size, limited statistical power, and the lack of gene expression data. Further investigation is required for a direct correlation analysis between expression and methylation. In addition, the confounding effects of contaminated tissues are also possibly influencing methylation levels.

## Conclusions

Our DNA methylation profiles served as a robust discriminator between asthmatics who are atopic or non-atopic for the *Dp* antigen. Functional studies will be valuable in identifying key genes or pathways that could serve as biomarkers or therapeutic targets.

## Competing interests

The authors declare that they have no conflicts of interest.

## Authors’ contributions

Conceived and designed the experiments: HDS and C-SP. Performed the experiments: Y-JK, T-HK. Analyzed the data: S-WP, J-SP, HSC. All authors have read and approved the final manuscript.

## Pre-publication history

The pre-publication history for this paper can be accessed here:

http://www.biomedcentral.com/1471-2350/14/39/prepub

## Supplementary Material

Additional file 1: Table S1 Gene ontology analysis of genes with different methylation states according to atopic status among BA groups. **Table S2.** Gene pathway analysis of gnens with different methylation states according to aoptic status among BA (Pathway Express:KEGG). **Table S3.** DNA methylation level of commonly used housekeeping genes. Click here for file
